# Catestatin and vasostatin concentrations in healthy dogs

**DOI:** 10.1186/s13028-016-0274-8

**Published:** 2017-01-03

**Authors:** Thanikul Srithunyarat, Ragnvi Hagman, Odd V. Höglund, Ulf Olsson, Mats Stridsberg, Supranee Jitpean, Anne-Sofie Lagerstedt, Ann Pettersson

**Affiliations:** 1 Department of Clinical Sciences, Swedish University of Agricultural Sciences, Box 7504, 75007 Uppsala, Sweden; 2 Department of Surgery and Theriogenology, Faculty of Veterinary Medicine, Khon Kaen University, Khon Kaen, 40002 Thailand; 3 Unit of Applied Statistics and Mathematics, Swedish University of Agricultural Sciences, Box 7032, 75007 Uppsala, Sweden; 4 Department of Medical Sciences, Uppsala University, 75185 Uppsala, Sweden

**Keywords:** Catestatin, Chromogranin A, Healthy dogs, Stress behavior visual analog scale, Vasostatin

## Abstract

**Background:**

The neuroendocrine glycoprotein chromogranin A is a useful biomarker in humans for neuroendocrine tumors and stress. Chromogranin A can be measured in both blood and saliva. The objective of this study was to investigate concentrations of and correlation between the chromogranin A epitopes catestatin and vasostatin in healthy dogs accustomed to the sample collection procedures. Blood and saliva samples were collected from 10 research Beagle dogs twice daily for 5 consecutive days, and from 33 privately-owned blood donor dogs in association with 50 different blood donation occasions. All dogs were familiar with sample collection procedures. During each sampling, stress behavior was scored by the same observer using a visual analog scale (VAS) and serum cortisol concentrations. Catestatin and vasostatin were analyzed using radioimmunoassays for dogs.

**Results:**

The dogs showed minimal stress behavior during both saliva sampling and blood sampling as monitored by VAS scores and serum cortisol concentrations. Few and insufficient saliva volumes were obtained and therefore only catestatin could be analyzed. Catestatin concentrations differed significantly and did not correlate significantly with vasostatin concentrations (*P* < 0.0001). Age, gender, breed, and time of sample collection did not significantly affect concentrations of plasma catestatin, vasostatin, and saliva catestatin.

**Conclusions:**

The normal ranges of plasma catestatin (0.53–0.98 nmol/l), vasostatin (0.11–1.30 nmol/l), and saliva catestatin (0.31–1.03 nmol/l) concentrations in healthy dogs accustomed to the sampling procedures were determined. Separate interpretation of the different chromogranin A epitopes from either saliva or plasma is recommended.

## Background

Chromogranin A (CgA) is a biomarker that is widely used in human medicine, but few studies of CgA have been reported in dogs [1–6]. Chromogranin A, an acidic glycoprotein that belongs to the Granin family, is stored in chromaffin granules and coreleased with catecholamines and neuroendocrine hormones from the adrenal medulla and sympathetic nerve endings when the sympathoadrenal-medullary system is activated [7, 8]. An active secretion of CgA into saliva in the submandibular gland has been found in humans [9], horses, and rats [10, 11]. Chromogranin A can be measured in saliva and blood in humans, pigs, cows, and dogs [4, 12–15]. Several bioactive peptides are derived from CgA degradation, including vasostatin, pancreastatin, catestatin, and serpinin [16–33]. These CgA bioactive peptides play different critical roles in the endocrine, cardiovascular, neurologic, and immune systems [21, 31, 34–36].

Chromogranin A is a reliable biomarker for diagnosing and monitoring treatment outcome and prognosis in humans suffering from neuroendocrine tumors [8, 37–39]. Chromogranin A has detected in the myocardium in several species and has multiple roles in cardiovascular homeostasis [40–42]. Chromogranin A and its derived peptides have shown promise as biomarkers for cardiovascular diseases such as hypertension, heart failure, myocardial infarction, and coronary syndromes [43–47]. Moreover, saliva CgA has been shown to be a sensitive biomarker for stress in humans and pigs [12, 14, 48–53]. Some evidence suggests that CgA could be useful as a biomarker for neuroendocrine tumors and stress also in dogs [1, 2].

In humans, saliva sampling is preferable to blood sampling for monitoring stress because the technique is noninvasive [54, 55] and humans can be informed of the procedure and deliver saliva samples by voluntary spitting into a container. To obtain spontaneous saliva samples in dogs, however, collection swabs must be intraorally placed. Although noninvasive, the saliva sample collection procedure itself may cause a stress reaction in dogs [56, 57]. To date, the stress response to saliva sampling in comparison with the stress response to blood sampling has not been evaluated in dogs. Stress evaluation can be performed by subjective and objective measurements, for example, scoring stress behavior using visual analog scale (VAS) scoring and measuring cortisol concentrations [58, 59].

Even though there are interspecies differences in CgA amino acid sequences, CgA can be measured in dogs [4]. A study on cross-reactivity between humans and dogs against different regions of the CgA molecule showed that CgA 17–38 (vasostatin) and CgA 361–372 (catestatin) could be measured using competitive radioimmunoassay (RIA) in dogs, whereas intact CgA could not [4]. Catestatin (CST) and vasostatin (VS), both CgA derived peptides, are bioactive. CST modulates catecholamine secretion (negative feedback) and has antihypertensive, antimicrobial, and cardiosuppressive effects [21, 22, 36, 60–63]. VS regulates plasma calcium, influences vasodilation, and has antimicrobial, and cardiosuppressive effects [17, 35, 42, 64–66]. Fundamental information concerning concentrations of CST and VS in healthy dogs of various breeds and gender that are accustomed to the sampling procedures is, however, lacking. The aims of this study were to investigate and compare concentrations of CST and VS in healthy dogs familiar with the collection procedures. In addition, we hypothesized that if CST and VS have similar halflives then the concentrations should not differ significantly.

## Methods

### Study design and ethical approval

This study was designed as a prospective clinical study in two parts; part one included destination bred research dogs, and part two included privately owned dogs admitted for routine blood donation. The study was approved by the Uppsala Ethical Committee (C301/12) and all dog owners were informed and gave their consent prior to participation, in accordance with Swedish legislation.

### Part one: research dogs

Three male and seven female research Beagle dogs (4–10 years old) were included. All dogs were determined as healthy by complete physical examination including mental status, general attitude, appetite, mucus membrane appearance, capillary refill time, rectal temperature, body weight, body condition score, hydration status, auscultation of heart and respiratory rate and sounds, abdominal palpation, and musculoskeletal system palpation. Dogs were familiar with being handled and routinely participated in practical teaching in the veterinary education program and were housed at the Research Animal Facility, Department of Clinical Sciences, Swedish University of Agricultural Sciences (SLU), Uppsala, Sweden.

#### Sampling of saliva and blood

Saliva and blood samples were collected twice daily for 5 consecutive days at the following times: (A) 6:30–7:30 am and (B) 1:00–2:00 pm, this time points were selected based on the results from previous pilot study [67, 68]. Saliva samples were collected using a swab size 10 × 30 mm (SalivaBio, Salimetrics, PA, USA) placed in the oral cavity for 1 min. The swab was then transferred into a 4.5-ml polypropylene cryotube (CryoPure Tubes, Sarstedt, Nümbrecht, Germany) and centrifuged at 3000 rpm (1401 g) for 15 min. The saliva deposited was stored at −70 °C until analysis of all samples.

Two ml of blood was collected from the distal cephalic vein using butterfly needles (BD Vacutainer, Becton-Dickson, Plymouth, United Kingdom) into lithium heparin tubes and clot activator tubes (Vacuette, Greiner Bio-One, Kremsmünster, Austria) and centrifuged at 3300 rpm (1695 g) for 5 min. The obtained heparinized plasma samples were freeze stored in cryotubes (Low Temperature Freezer Vials, VWR, Stockholm, Sweden) at −70 °C until analysis of all samples within a maximum of 11 months storage time. The saliva sampling was performed within 5 min prior to blood sampling.

#### Visual analog scale (VAS)

Visual analog scale scoring was performed during each saliva and plasma sampling occasion to score the subjective stress behavior on a plain 100-mm line. The pre-established subjective criteria used in this study to determine stress behaviors during saliva and blood sampling are given in Table 1 [69]. All sampling procedures were performed by the same two veterinarians and the sampling stress behavior VAS scores determined by one observer (TS).

**Table 1:** Pre-established subjective criteria used to determine stress behaviors using visual analog scale

Stress intensity	No stress	Mild stress	Moderate stress	Severe stress
A. Criteria used for saliva sampling				
Criteria		Turns head away Spits Lifts paw Moves away	Turns head away Spits Lifts paw Moves away Avoids sampling Lifts lip Shakes Raises hair Growls	Turns head away Spits Lifts paw Moves away Avoids sampling Lifts lip Shakes Raises hair Growls Not able to sample Not able to touch Bites Attacks
B. Criteria used for blood sampling				
Criteria		Withdraws leg Moves away	Withdraws leg Moves away Avoids sampling Lifts lip Shakes Raises hair Growls	Withdraws leg Moves away Avoids sampling Lifts lip Shakes Raises hair Growls Not able to sample Not able to touch Bites Attacks

This stress behavior criteria are modified from Norling et al. [69]

### Part two: blood donor dogs and donation routines

In total, thirty-three privately-owned dogs, twenty-four males and nine females, aged from one to eight years, of fourteen different breeds (Boxer, Bernese Mountain Dog, Collie, Dalmatian, Flat Coated Retriever, German Shepherd Dog, Golden Retriever, Great Dane, Greyhound, Labrador Retriever, Leonberger, Shorthaired Pointer, White Shepherd, and Mixed Breed), were included in the study. All dogs that routinely donated blood at the University Animal Hospital (UDS), SLU, Uppsala, Sweden during April 2014, and from September to February 2015 were included. All dogs underwent a complete physical examination (as used for the research dogs) and blood samples were obtained from the distal cephalic vein and evaluated for health control purposes. Hematology and biochemistry (creatinine, alanine aminotransferase (ALT), alkaline phosphatase (ALP), total protein, and albumin) were measured using in-house equipment (IDEXX ProCyte Dx and IDEXX Catalyst Dx, IDEXX Laboratories, Maine, USA). In all dogs, positive and negative DEA 1.1 blood type (Quick Vet, Scandinavian Micro Biodevices ApS, Farum, Denmark) and presence of vector borne diseases including *Anaplasma phagocytophilum, Anaplasma platys, Borrelia burgdorferi, Ehrlichia canis, Ehrlichia ewingii, and Dirofilaria immitis* (Snap 4DX tests, IDEXX Laboratories, Maine, USA) were determined. Dogs with antibodies against *Anaplasma phagocytophilum, Anaplasma platys, Borrelia burgdorferi, Ehrlichia canis, and Ehrlichia ewingii* were considered healthy if no signs of active infection was present when examined, whereas dogs with positive antigens of *Dirofilaria immitis* were considered infected. Only healthy dogs were allowed to donate blood, and were enrolled for routine donation every 3–4 months. All dogs included in the present study were familiar with the sampling procedures and needed no sedation during collection. Dog owners were present throughout procedures.

#### Sampling of saliva and blood

Heparinized plasma and serum samples remaining after the routine hematology and biochemistry analysis were used for the study. In total, seventeen dogs donated blood on one occasion whereas fifteen donated twice and one donated three times on different occasions (more than 3 months interval) resulting in fifty separate samplings. All blood samples were collected by the same two certified veterinary nurses. Saliva sampling was performed by TS using the same criteria as in research dogs. In contrast to the sampling in research dogs, for practical reasons, blood and saliva sampling was performed on variable times between 8:00 am–2:00 pm, and the order in which the samples were collected was randomized with an interval between saliva and blood sampling of less than 10 min. All samples were handled and stored in the same manner as described for research dogs.

#### Visual analog scale (VAS)

Visual analog scale scoring was performed by TS using the same criteria as in research dogs.

### Analysis of catestatin and vasostatin

Competitive radioimmunoassay (RIA) was used for measuring CST and VS. All heparinized plasma samples were analyzed in duplicates at the Clinical Chemistry Laboratory, Uppsala University Hospital, Uppsala, Sweden as previously described [4, 70]. This method has been developed for both tissue and circulation and used for measuring CgA in humans. The detection limit is 0.01 nmol/l for plasma CST and VS and 0.04 nmol/l for saliva CST and the total coefficient of variance (CV) was <10%. The overall CV in the present study was <10%. For each analysis, 300 µl saliva and 100 µl plasma were required. The saliva sample volume obtained was insufficient for analysis of saliva VS.

### Cortisol analysis

Serum samples were analyzed for cortisol concentrations in duplicate using a solid-phase competitive chemiluminescent enzyme immunoassay (Immulite 2000, Siemens, Erlangen, Germany) at Clinical Chemistry Laboratory, UDS. The intraassay CV was <5%.

### Statistical analysis

In all analysis of CST and VS, diagnostic plots were used to assess normality and homoscedasticity. Because the distributions of residuals for CST and VS data appeared skewed, these data were log transformed (natural log) prior to analysis. After transformation, no apparent deviations from normality and homoscedasticity could be detected.

In all analysis, post hoc comparisons of least squares means were adjusted for multiplicity using Tukey’s method. Results were considered significant when *P* < 0.05. Most analysis were made using the mixed procedure of the SAS package 2014, but other procedures for basic statistics, Proc Univariate, Proc Corr and Proq Freq, were also used. Normal range was calculated using percentile 2.5–97.5 of log transformed data and back-transformed to the original scale.

#### Research dog data

Because several measurements were made in each dog, mixed linear models [71, 72] were used for the analysis. The fixed part of the models included the variables “category” with three levels (plasma CST, plasma VS, and saliva CST); day (1–5); gender (male or female); time of day (am or pm), and interactions between these factors. The random part of the model included dog, dog * day and dog * category.

The relations between the three measurements (categories) were modeled by allowing the R-side correlations among them to be an unstructured correlation matrix [71, 73]. This corresponds to using multivariate analysis of variance (MANOVA) model, but still allows for inclusion of random effects in the model.

#### Blood donor dog data

For the blood donor dog data, the same categories as for the research dog data were used. Because the same dog could have data for one, two or three donation occasions, mixed models (*ibid*) were also used for these data.

Several models were tried. The fixed part of the models included category as above. Moreover, different background variables for the dogs (gender, age, and breed) and site (plasma and saliva) were tested. The R-side correlations were modeled as for the research dogs. Random effects were dog, dog * site and dog * site * variable.

#### Comparisons between blood donor dogs and research dogs

The two data sets were collected in different ways. To allow comparisons between the groups, mean values were calculated, for all variables, for each dog. This led to a simple data set where comparisons between groups could be made using one-way ANOVA, or, equivalently, using two-sample t tests. These data sets were also used for calculating correlations between different variables.

## Results

The mean ± SD age and body weight was 7.5 ± 2.6 years and 14.3 ± 1.2 kg in research dogs and 3.7 ± 2.0 years and 36.2 ± 9.8 kg in blood donor dogs. From the 50 blood donation occasions, 48 plasma and 40 saliva samples were obtained. Due to insufficient volumes remaining in some cases, plasma CST was analyzed in 39 of the 48 plasma samples, plasma VS in 44 of the 48 plasma samples and saliva CST in 40 of the 40 saliva samples. In the research dogs, plasma CST, plasma VS, and saliva CST could be analyzed in all samples collected (100). Mean ± SD values of serum cortisol were 39.9 ± 6.1 nmol/l in research dogs and 65.8 ± 28.2 nmol/l in blood donor dogs. Mean ± SD values of plasma CST, plasma VS, saliva CST, blood and saliva sampling stress behavior VAS scores from research and blood donor dogs are illustrated in Table 2. No significant differences were found between research and blood donor dogs. The normal ranges of plasma CST, plasma VS, and saliva CST in this study was 0.53–0.98, 0.11–1.30, and 0.31–1.03 nmol/l, respectively.

**Table 2:** Mean ± SD of plasma catestatin, vasostatin, and saliva catestatin and sampling stress behavior score

Parameters	Research dogs (n = 10)	Blood donor dogs (n = 33)
Plasma catestatin (nmol/l)	0.81 ± 0.08^a^	0.76 ± 0.10^a^
Plasma vasostatin (nmol/l)	0.57 ± 0.55^b^	0.44 ± 0.39^b^
Saliva catestatin (nmol/l)	0.83 ± 0.12^a^	0.64 ± 0.21^c^
Blood sampling stress behavior VAS score (mm)	8.9 ± 10.5^a^	19.1 ± 17.3^a^
Saliva sampling stress behavior VAS score (mm)	11.1 ± 7.8^a^	21.2 ± 16.7^a^

In research dogs, plasma vasostatin concentrations differed significantly from plasma and saliva catestatin concentrations. In blood donor dogs, plasma catestatin, vasostatin, and saliva catestatin differed significantly. Blood and saliva sampling stress behavior VAS scores did not differ significantly between both dog groups

^a,b,c^Different letters within each column of concentration and stress behavior VAS score indicate significant differences using Tukey’s method adjustment (*P* < 0.05)

Plasma CST, plasma VS, and saliva CST concentrations did not correlate significantly in any of the groups of dogs. In research dogs, plasma VS concentrations differed significantly from plasma and saliva CST concentrations (*P* < 0.002). In blood donor dogs, plasma CST, VS, and saliva CST differed significantly (*P* < 0.0001). Plasma CST, plasma VS, and saliva CST concentrations did not differ significantly between different collection times in research dogs. Plasma and saliva CST concentrations did not differ significantly when compared between different days of collection. Plasma CST, plasma VS, and saliva CST concentrations did not differ significantly between ages, genders, and breeds in both dog groups. Stress behavior VAS scores were low in all dogs and did not differ between sampling methods, ages, genders, breeds, collection time or day.

In the blood donor dogs, all hematology and blood chemistry results were deemed acceptable for blood donation. Nineteen dogs were positive for DEA 1.1 and 14 negative for DEA 1.1. Plasma and saliva CST and VS did not differ significantly between blood groups. None of the blood donor dogs had positive antigens of *Dirofilaria immitis.* Six dogs had antibodies against *Borrelia burgdorferi* without clinical signs of disease. Plasma CST, VS, and saliva CST concentrations did not differ significantly between positive and negative *B. burgdorferi*.

## Discussion

This is the first study in dogs that investigates concentrations of and correlations between CST and VS in healthy dogs familiarized with a sample collection procedure. The concentration values and ranges reported here can be used as reference ranges for plasma CST, plasma VS, and saliva CST concentrations in healthy dogs when analyzed by RIA. Our findings will be useful in future studies on the role and possibilities of using CST and VS as biomarkers in dogs. In a previous study of dogs with pyometra, using the same RIA as in the current study, the reported serum CST concentrations in healthy control dogs were higher than reported here [5]. This difference between studies could be due to different familiarity to the handling techniques, and sample storage time. Although CgA has been reported to be heat stable and the concentrations are stable through freeze and thaw cycle in humans and pigs [15, 74, 75], studies on CST and VS in dogs are lacking. In contrast to the previous study, the dogs included in our study were all well accustomed to the sampling procedures prior to sample collection, and showed minimal stress behaviors as monitored by stress behavior VAS scoring and serum cortisol concentrations [76].

Although both CST and VS are derived from CgA, the concentrations of plasma CST and VS in this study reflect both the intact CgA molecule and the two respective degradation derived peptides. The significant differences in concentrations seen in the present study may be because the peptides have different functions and clearance rates. The CgA derived peptides might also be secreted differently into saliva and blood [13] which could contribute to our finding that CST concentrations were different in saliva and plasma in the blood donor dogs. Nevertheless, plasma CST, plasma VS, and saliva CST concentrations did not correlate significantly in both dog groups. The concentrations of plasma CST, plasma VS, and saliva CST also did not vary by age, gender, or breed in either group of dogs. The results of the present study show that it is crucial to evaluate different CgA peptides individually, and with regard to whether measurements were made in plasma or saliva because otherwise the results are not comparable.

Saliva sampling is preferable in humans because it is less invasive than blood sampling [54, 55]. In dogs, however, stress behaviors during saliva and blood sampling have not previously been evaluated and prolonged sampling time could induce stress [56, 57]. All dogs, in the present study were well accustomed to the sampling procedures and exhibit minimal stress levels as shown by stress behavior VAS scores and serum cortisol concentrations. However, in order to avoid inducing stress behavior we limited the time for saliva sampling to 60 s. We also chose not to pharmacologically induce saliva secretion because this may affect the secretion of neuroendocrine peptides. In the present study, there was no significant difference in the dogs’ acceptance of blood or saliva sampling as monitored by the stress behavior VAS. Our findings indicate that saliva sampling is unpredictable and that for our research purposes, blood sampling is a better choice.

In human studies, saliva CgA has been used for evaluating stress [12, 49–51]. In humans and pigs, active CgA secretion from the mandibular salivary gland has been found [9, 12, 14], however, little is known about CgA secretion in dogs. If similar active secretion also occurs in dogs, analysis of CST or VS in saliva may still be useful for monitoring stress levels. However, the sampling techniques need to be improved to ensure sufficient sample volumes are obtained without undue stress or pharmacological intervention.

Ten research Beagle dogs were included in the study to investigate whether concentrations of CST and VS varied over time in the same individual. In a previous pilot study, using the same RIA, five research Beagle dogs were sampled four times daily between 6:30 am and 3:00 pm, saliva CST was increased in samples collected between 6:30 and 8:00 am [67, 68]. In addition, CgA in saliva has been found to be increased in early morning samples (7:00 am) in humans [13, 77, 78]. In our study, there was no significant difference in the concentrations of plasma CST, VS and saliva CST between time of day, which is in agreement with a previous study that used an ELISA for human CgA 344–374 amino acid sequence for saliva samples in dogs [3]. On the other hand, a circadian variation in CgA has been found in plasma and saliva in humans [13, 79]. However, as stated previously comparisons between different species and studies on different peptides should be made with caution.

The bioactive peptides of CgA have shown promise as prognostic and diagnostic biomarkers for neuroendocrine tumors, cardiac disease, burn trauma and stress in humans [12, 35, 80–82]. The main focus of this study was to investigate the concentrations of and correlation between CST and VS in healthy dogs familiar with the collection procedures. Further studies are warranted to investigate whether CST and VS can be used as biomarkers for neuroendocrine tumors, cardiovascular diseases, and stress in dogs.

## Conclusions

Concentrations of plasma CST (0.53–0.98 nmol/l), plasma VS (0.11–1.30 nmol/l), and saliva CST (0.31–1.03 nmol/l) in healthy dogs accustomed to the sampling procedures were determined. The concentrations of plasma CST, plasma VS, and saliva CST significantly differed and were unaffected by age, gender, breed, and time of sampling. No significant correlation between plasma CST and VS, as well as saliva CST could be found indicating that separate interpretation of the different CgA epitopes from either saliva or plasma is mandatory.

## Abbreviations

CgA: chromogranin A
CST: catestatin
ELISA: enzyme-linked immunosorbent assay
RIA: radioimmunoassay
RPM: revolutions per minute
VS: vasostatin
VAS: visual analog scale
